# Nutritional Profile of Phytococktail from Trans-Himalayan Plants

**DOI:** 10.1371/journal.pone.0083008

**Published:** 2013-12-23

**Authors:** Priyanka Dhar, Amol B. Tayade, Jatinder Kumar, Om P. Chaurasia, Ravi B. Srivastava, Shashi B. Singh

**Affiliations:** 1 Defence Institute of High Altitude Research, Defence Research and Development Organisation, Leh-Ladakh, Jammu and Kashmir, India; 2 Defence Institute of Physiology and Allied Sciences, Defence Research and Development Organisation, Timarpur, Delhi, India; University of Sassari, Italy

## Abstract

We estimated the nutritive value, vitamin content, amino acid composition, fatty acid content, and mineral profile of a phytococktail comprising sea buckthorn (*Hippophae rhamnoides*), apricot (*Prunus armeniaca*), and roseroot (*Rhodiola imbricata*) from trans-Himalaya. The free vitamin forms in the phytococktail were determined by rapid resolution liquid chromatography/tandem mass spectrometry (RRLC-MS/MS). Vitamin E and B-complex vitamins were detected as the principle vitamins. Reversed-phase high performance liquid chromatography (RP-HPLC) with pre-column derivatization was used for identification and quantification of amino acids. Eight essential and eleven non-essential amino acids were quantified, and the content ranged between 76.33 and 9485.67 µg/g. Among the essential amino acids, L-methionine, L-phenylalanine, L-lysine, L-leucine, and L-histidine were found to be the dominant contributors. We also quantified the fatty acids in the phytococktail by using gas chromatography coupled with a flame ionization detector (GC-FID) with fatty acid methyl esters (FAMEs) derivatization. The analysis revealed the presence of 4 major fatty acids contributing to the total lipid content. Palmitic acid was found to be the rich source of saturated fatty acid (SFA) and constituted ∼31% of the total lipid content. Among the unsaturated fatty acids (UFAs), palmitoleic acid (43.47%), oleic acid (20.89%), and linoleic acid (4.31%) were prominent. The mineral profiling was carried out by inductively coupled plasma optical emission spectrometer (ICP-OES), and it was found to contain a number of important dietary mineral elements. The harsh climatic conditions, difficult terrain, and logistic constraints at high altitude regions of Indian trans-Himalayan cold desert lead to the scarcity of fresh fruits and vegetables. Therefore, the source of multiple vitamins, essential amino acids, fatty acids, and dietary minerals from the phytococktail would provide great health benefit in the stressful environment and could be used as a high value nutritional supplement.

## Introduction

Plant-derived foods such as fruits, vegetables, grains, oils, nuts etc. have become an essential part of human diet for their beneficial health promoting effects. The implication for the anticipatory and therapeutic utility of nutrients and dietary supplements from plant based phyto products are profound and these are one of the promising therapeutic means available to avert, delay, alter or eradicate various signs and symptoms of nutrition deficiency disorders [1], [2]. The Indian sub-continental diets, in particular, also consist of a diverse range of foods such as cereals, pulses, green leafy vegetables, roots, tubers, fruits, oil seeds, spices and condiments [3]. These botanical foods provide a large number of vital dietary components such as vitamins, antioxidants, amino acids, fatty acids, minerals etc. are essential for human health.

Vitamins are characterized by a cluster of both chemically and analytically heterogeneous compounds that may exist in numerous chemically diverse but biologically inter convertible forms. These are one of the important essential micronutrients for human nutrition and a significant part of world’s population is still prone to the health threats linked with low micronutrient ingestions even after taking balanced diet [4], [5]. Therefore, intake of dietary supplements with optimum vitamin content is a useful strategy to maintain proper health. Vitamins can be broadly classified in two major groups, water-soluble and fat-soluble vitamins. Water-soluble vitamins include B group vitamins *viz*. thiamine (B_1_), riboflavin (B_2_), niacin (B_3_), pantothenic acid (B_5_), pyridoxine (B_6_), biotin (B_7_), folic acid (B_9_), cyanocobalamin (B_12_) and ascorbic acid (C) while the fat-soluble vitamins are retinol (A), ergocalciferol (D_2_), tocopherol (E) and phylloquinone (K_1_). These vitamins play a number of vital functions in metabolism and can cause health problems when they are either deficient or surplus in the human body [6]. Various analytical techniques such as spectrometric assays [7], volumetric assays [8], fluorimetry [9], chemiluminiscence [10], microbiological assays [11], capillary electrophoresis [12], thin-layer chromatography [13], high-performance liquid chromatography (HPLC) [14], HPLC-mass spectrometry (HPLC-MS) and liquid chromatography-tandem mass spectrometry (LC-MS/MS) [15], [16] have been reported for analysis of vitamins in different biological samples. Among these techniques rapid resolution liquid chromatography coupled with tandem mass spectrometric detection (RRLC-MS/MS) is considered to be a powerful method for determination of multiple vitamins in different foods and food products, nutraceuticals and pharmaceutical preparations.

Occurrence of amino acids in nature is either in free form or as linear chains in peptides and proteins. In the study of the composition of proteins, foods, feedstuffs and other biological preparations analysis of amino acids has a significant role. A total of 22 proteinogenic amino acids are found in nature of which 20 genetically encoded protein amino acids are the building blocks of proteins and vital constituents of all biological systems. Most of the proteins in multicellular organisms are based on L-amino acids that exert great influence in both human and animal nutrition and possess strong therapeutic potential [17], [18]. Human beings are able to synthesize 11 of the 20 amino acids encoded by the universal genetic code and the other 9 amino acids, *viz.* histidine, isoleusine, lysine, leucine, methionine, phenylalanine, threonine, tryptophan and valine are called essential amino acids. These are so named because they must be consumed in the diet to maintain optimum cellular and physiological functions. Therefore, intake of amino acids in the form of nutraceutical supplements could have beneficial effects to human body. It can produce incredible effects to counter nutrient deficiency disorders and many other disease conditions when taken in concentrated form. There are a large number of advanced strategies like capillary electrophoresis-mass spectrometry (CE-MS), gas chromatography-mass spectrometry (GC-MS), liquid chromatography-mass spectrometry (LC-MS) etc. have been used for determination of amino acids in biological systems [19]–[22]. In the pharmaceutical industry enantiomeric separation of amino acids has gained extreme interest in recent time. The separation of amino acids and other chiral compounds can be achieved by two major approaches. The first strategy is an *indirect method*, based on the formation of diastereomers by the reactions of amino acids with a chiral derivatizing agent (CDA) and separation of the diastereomeric derivatives on an achiral stationary phase. The second strategy is a *direct method*, based on the formation of diastereomers on a chiral stationary phase (CSP) or with a chiral selector on an achiral stationary phase in a mobile phase. The *indirect method* is best suited technique for chromatographic enantioseparation of amino acids with a number of advantages [23]. Analysis of amino acids present in foods is usually performed after their derivatization. Reversed phase high performance liquid chromatography (RP-HPLC) technique with pre-column derivatization is generally preferred because of the short time, simple instrumentation and cost-effectiveness. The pre-column derivatization of amino acids are achieved by a number of distinct reagents *viz.* phenylisothiocyanate (PITC); o-phthalaldehyde (OPA); 9-fluorenylmethyl-chloroformate (FMOC-Cl); 1-fluoro-2,4-dinitrobenzene; 1-fluoro-2,4-dinitrophenyl-5-L-alanine amide; dansyl-chloride etc. [24]. Among these, the PITC reagent is widely used for amino acid analysis [25], [26].

Fatty acids are aliphatic monocarboxylic acids that function as the building blocks of lipids. There are two broad groups of fatty acids *viz.* saturated fatty acids (SFA, without any double bond) and unsaturated fatty acids (UFA, with double bonds) present in nature. The UFAs are further classified into monounsaturated fatty acids (MUFA) and polyunsaturated fatty acids (PUFA). Fatty acids have distinct biological activities and provide numerous health promoting effects [27]. Plants contain lipids that are the main sources of PUFAs. The omega-6 (n-6) and omega-3 (n-3) fatty acids are the two major PUFAs derived from α-linolenic acids (18∶3 n3) and linoleic acid (18∶2 n6), which are essential dietary components for animal and human diet to uphold optimum health [28]. Precise determination of fatty acid profiles in nutritional, epidemiological and clinical research has become an essential parameter. Gas chromatography coupled with flame ionization detection (GC-FID) is a rapid and efficient method for fatty acid profiling in biological materials [29]. In this process, the less volatile fatty acids are converted into more volatile derivatives called fatty acid methyl esters (FAMEs) prior to GC analysis.

India has rich plant biodiversity with highly divergent ecosystems and significant altitudinal variations ranging from sea level to the highest ranges of the Himalaya and other allied factors. The high altitude region of trans-Himalayan cold desert possesses adverse climatic conditions for human survival and there is ample evidence that in this unfavorable environment, sustained energy deficit, malnutrition, vitamin and mineral deficiency and metabolic disorders could occur due to alteration in physiological function [30], [31]. However, the Himalaya also has the remedy for these problems in its diverse flora and fauna. The Himalayan plants are widely used in traditional system of medicine both as prophylactics and therapeutics for high altitude maladies. A number of natural products from the plants of this region have also been formulated from institute [32], [33]. However, there is still a need to formulate a phytococktail with high adaptogenic properties that could be used for facilitating performance of troops and people going to high altitude. We aimed to formulate the phytococktail comprising of sea buckthorn (*Hippophae rhamnoides* L., Elaeagnaceae), apricot (*Prunus armeniaca* L., Rosaceae) and roseroot (*Rhodiola imbricata* Edgew., Crassulaceae) which could be capable of providing additional physiological benefits and meeting basic nutritional requirements in this extreme environment. The medicinal and therapeutic potential of the three plants have been extensively studied by previous researchers and these were reported to possess diverse bioactive constituents that ameliorate chronic diseases [34]–[38]. In our recent study, we have reported the antioxidant capacities and chemical composition of this phytococktail [39], [40]. However, the nutritional and mineral profile of the phytococktail remains to be elucidated. Hence, our aim was to assess the nutritional profile of the phytococktail developed from the trans-Himalayan plants. We have estimated the nutritive value, vitamin content, amino acid composition, fatty acid content and mineral profile of the phytococktail by standard techniques.

## Materials and Methods

### Ethics Statement

All necessary permits were obtained for the described field studies and plant collection. The permit was issued by Dr. B. Balaji (IFS), Divisional Forest Officer, Leh Forest Division, Jammu & Kashmir, India.

### Chemicals

Reference standards of fat soluble vitamins: *trans*-retinol (A), ergocalciferol (D_2_), D-α-tocopherol (E) and phylloquinone (K_1_); water soluble vitamins: thiamine hydrochloride (B_1_), riboflavin (B_2_), nicotinic acid (B_3_), nicotinamide (B_3_), D-pantothenic acid (B_5_), pyridoxine hydrochloride (B_6_), D-biotin (B_7_), folic acid (B_9_) and cyanocobalamin (B_12_) were procured from Sigma-Aldrich (Sigma-Aldrich, St. Louis, MO, USA). Triethylamine (TEA), phenylisothiocyanate (PITC) and amino acid standards, sodium hydrogen phosphate (Na_2_HPO_4_), sodium sulphate (Na_2_SO_4_), boron trifluoride (BF_3_), sodium acetate trihydrate (CH_3_COONa.3H_2_O), toluene and phosphoric acid (H_3_PO_4_) were also purchased from Sigma-Aldrich. HPLC grade acetonitrile, ethanol, methanol, dietyl ether, 2-propanol, n-hexane, acetyl-chloride, petroleum ether, chloroform and glacial acetic acid were purchased from Merck (Merck KGaA, Darmstadt, Germany). The ultra pure water was prepared by a Milli-Q purification system (Millipore Corp., Bedford, MA, USA). Potassium hydroxide (KOH), sodium hydroxide (NaOH), potassium sodium tartrate (NaK tartrate), sodium carbonate (Na_2_CO_3_), copper (II) sulphate pentahydrate (CuSO_4_.5H_2_O), anthrone, glucose, hydrogen peroxide (H_2_O_2_), hydrochloric acid (HCl), sulphuric acid (H_2_SO_4_) and nitric acid (HNO_3_) were of analytical grade purchased from Merck (MERCK, KGaA, Darmstadt, Germany). The chemicals and reagents used were of HPLC and/or analytical grades. Commercial reference FAMEs standard mixture (Supelco 37-Component FAME Mix, 47885-U, Supelco, Bellefonte, PA, USA) containing methyl esters of fatty acids ranging from C4 to C24, including key monounsaturated and polyunsaturated fatty acids were used. Nitrogen (industrial grade) was obtained from Sigma Gases & Services (Delhi, India) and used without further purification. All solutions were passed through 0.45 µm Teflon membrane filters (MetaChem, Torrance, CA, USA) prior to analyses. The pH value of the solution was equilibrated with HI 8424 Portable pH/mV/°C meter (HANNA Instruments Inc., RI, USA) regularly calibrated with National Bureau of Standards (NBS) buffer solutions.

### Plant Materials and Phytococktail Preparation

*H. rhamnoides* berries were collected from the Choglamsar village of Leh, Ladakh, India [altitude 3500 m above sea level (ASL), latitude 34°6′38.9664″N, longitude 77°35′10.3992″E], in October, 2010. Mechanical pulping of berries yielded raw pulp that was 50% of the fruit weight. The pulp was then lyophilized to obtain a dry mass. The fruits of *P. armeniaca* (Halman variety) were collected from the apricot field gene bank of Defence Institute of High Altitude Research, Leh (altitude 3500 m ASL, latitude 34°8′16.119″N, longitude 77°34′19.2216″E), in September, 2010. Pulping was done by mechanical pulper and yield of pulp was 70% of the total fresh fruit weight. The pulps of sea buckthorn and apricot were then freeze dried to obtain dry powder. Roots of *R. imbricata* (roseroot) were collected from the Changthang valley of trans-Himalayan region (Chang-La Top, altitude 5330 m ASL, latitude 34°2′49.812″N, longitude 77°55′49.7778″E) of India in the month of October, 2010. The plant samples were washed thoroughly and roots were cut into small pieces, shade dried at room temperature for 15 days, finely powdered and used for extraction. The root powder was taken for extraction in 80% ethanol by Soxhlet apparatus (Borosil GlassWorks Limited, Worli, Mumbai, India). The ethanolic fraction was concentrated under reduced pressure at 40°C and lyophilized to obtain dry extract. Preparation of the phytococktail was carried out as described previously [39], [40].

### Vitamin Content Analysis

Determination of vitamin content in the phytococktail was achieved according to the recent report from our institute [38].

#### Chromatographic apparatus & RRLC-MS/MS method details

Detection and quantification of fat and water soluble vitamins were performed using the Agilent 1200 Series Rapid Resolution Liquid Chromatography (RRLC) Binary modules interfaced to the Agilent 6410 Triple Quadrupole (QQQ) RRLC-MS/MS (G6410A, Agilent Technologies, Santa Clara, CA, USA) with HPLC-Chip Cube. The RRLC system was equipped with Agilent 1200 series vacuum micro degasser (G1322A), Agilent 1200 series binary pump system SL (G1312B), Agilent 1200 series High Performance Autosampler (HPALS) SL (G1367C) and Agilent 1200 series Thermostated Column Compartment (TCC) SL (G1316B). The Triple Quadrupole Mass Spectrometer (QQQ-MS) was equipped with an electrospray ionization (ESI) probe. The RRLC system was controlled by Agilent ChemStation module. MassHunter versions B.02.01 and B.03.01 were used for data acquisition, qualitative, and quantitative analysis.

RRLC-MS/MS determination of fat and water soluble vitamins was carried out on Agilent Poroshell 120 EC-C18 Narrow bore, 2.1×100 mm, 2.7 µm particle size column (p/n 695775-902). Column temperature was maintained at 35°C. The mobile phase comprised of 0.1% formic acid (HCOOH) in water with 10 mM ammonium formate (NH_4_COOH) (mobile phase A) and 0.1% HCOOH in methanol with 10 mM NH_4_COOH (mobile phase B). Flow rate was 0.3 ml/min. The RRLC gradient elution condition has been depicted in Table 1. The injection volume was 5 µl. A 550 bar pressure was maintained. The autosampler compartments were thermostated at 5°C. To identify and quantify the fat and water soluble vitamins, QQQ-MS was operated in the positive ESI mode, using Agilent G1948B ionization source. Capillary voltage was set at 2500 V. Source temperatures for fat and water soluble vitamins were set at 325°C and 350°C, respectively. Drying gas flow was 8 l/min with nebulizer pressure set at 45 and 50 psi for fat and water soluble vitamins respectively. Multiple reactions monitoring (MRM) scan was applied for scanning and acquiring MS data and quantitation.

**Table 1:** RRLC gradient^a^ elution program for the separation of fat- and water-soluble vitamins on Agilent Poroshell 120 EC-C18 reverse phase column.

Time (min)	Flow rate(ml/min)	Solvent A^b^(%, v/v)	Solvent B^c^(%, v/v)
Fat-soluble vitamins			
0	0.3	10	90
3	0.3	10	90
4	0.3	0	100
17	0.3	0	100
18	0.3	10	90
25	0.3	10	90
Water-soluble vitamins			
0	0.3	90	10
8	0.3	45	55
10	0.3	45	55
11	0.3	90	10
18	0.3	90	10

^a^ Total run time = 25 and 20 min, for fat- and water- soluble vitamins, respectively; post time = 5 min for fat- and water-soluble vitamins.

^b^ A = 0.1% formic acid in water +10 mM ammonium formate.

^c^ B = 0.1% formic acid in methanol +10 mM ammonium formate.

#### Preparation of standard solutions

Standards of vitamin B_1_, B_3_ (nicotinamide and nicotinic acid), B_5_, B_6_, B_7_ and B_12_ were prepared by accurately weighing 1 mg of the respective vitamin standards in 1 ml of MilliQ water to form stock solutions of 1 mg/ml of each vitamin. Vitamin B_2_ and B_9_ had limited solubility in water. Hence, the stock solution of vitamin B_2_ was prepared in 5 mM KOH. Stock solution of vitamin B_9_ was prepared in 20 mM KHCO_3_ instead of MilliQ water [41]. A standard mixture containing 9 water soluble vitamins *viz*. thiamine (B_1_), riboflavin (B_2_), nicotinic acid (B_3_), nicotinamide (B_3_), D-pantothenic acid (B_5_), pyridoxine (B_6_), D-biotin (B_7_), folic acid (B_9_) and cyanocobalamin (B_12_) was diluted in water: methanol (90∶10, v/v) with 10 mM ammonium formate and 0.1% formic acid to the following concentrations: 10, 50, and 100 ppb. One milligram each of vitamin A, D_2_ and E were accurately weighed and dissolved in 1 ml of methanol to form 1 mg/ml stock solution of each vitamin. Vitamin K_1_ was prepared in acetone. The stock standard solutions were stored at 4°C for further analysis. A standard mixture containing 4 fat soluble vitamins *viz*. retinol (A), ergocalciferol (D_2_), α-tocopherol (E) and phylloquinone (K_1_) was diluted in methanol: water (90∶10, v/v) with 10 mM ammonium formate and 0.1% formic acid to the following concentrations: 100, 500, and 1000 ppb [42].

#### Sample preparation and extraction of vitamins

Water soluble vitamins were extracted by a combination of acid and enzymatic hydrolysis [43]–[45]. Dried phytococktail powder (1 g) was suspended in 25 ml of 0.1 N HCl and then autoclaved at 100°C for 20 min. It was then allowed to cool and the pH was adjusted to 4.0 with 2 N sodium acetate. After that 2 ml of 2% Clara-diastase suspension was added to the sample and it was submitted to enzymatic digestion for 18 h at 37°C. Then it was cooled to ambient temperature and the volume was adjusted to 1 l with MilliQ water, followed by filtration through a 0.45 µm glass microfiber membrane (Whatman® GD/X) and the filtrate was collected for RRLC-MS/MS analysis [46]. Sample preparation procedures were carried out under dim light and samples were kept in the amber vials.

Extraction of fat soluble vitamins was carried out with 1 g of phytococktail powder. It was transferred into 10 ml volumetric flask and then 8 ml of methanol-dichloromethane (1∶1, v/v) containing 0.1% BHT was added to it. After 15 min of ultrasonic extraction, methanol-dichloromethane was added to the mark and filtered through a 0.45 µm glass microfiber membrane. The prepared sample solution was stored in dark till further analysis [41].

### Amino Acid Analysis

The amino acid content of the phytococktail was determined using RP-HPLC with pre-column PITC derivatization [47], [48].

#### RP-HPLC system

The RP-HPLC system was equipped with a Shimadzu Class VP Binary pump LC-10AT_VP_, DGU-14A On-Line Degasser, 2 µl in-line precolumn filters, 100 µl Semi-Micro Gradient Mixer, SIL-10A_VP_ Auto sampler, CTO-10A_VP_ column temperature Oven and SPD-10Avp UV/VIS Detector. All the components of the system were controlled using SCL-10Avp System Controller with PC-55N A/D option board for SCL and a Model 3394A integrator (Hewlett-Packard, Avondale, PA). Reversed phase C-18 column (5 µm, 150×4.6 mm) (Pickering Laboratories, Inc., Mountain View, California, USA) was used for the separation of amino acids. A 30×4.6 mm i.d. guard column of the same material was also employed. Data acquisitions were done using Windows® 2000 Data Station and CLASS-VP™ Version 6.13 software with 0.005% minimum detection limit (MDL).

#### Standard preparation

Individual 21 amino acid standards (10 mg) were prepared by dissolving in 10 ml distilled water. A working standard mixture was prepared by diluting the intermediate stock standard solution to 100 µg/ml. Two hundred microliter of this standard solution was derivatized as discussed in the next section. The derivatized standard solution was further diluted with distilled water up to 500 µl to prepare a final concentration of each amino acid of 40 µg/ml in the working standard solution. The solution was filtered and stored at −20°C for further HPLC analysis.

#### Extraction of amino acids

One gram of phytococktail was weighed into a 25×150 mm hydrolyzed tube, aliquot (15 ml) of 6 N HCl was added, purged with nitrogen for 30 sec and the tube was sealed immediately with teflon coated cap. The tube was placed in the oven at 110°C for 24 h to hydrolyze the protein completely [49], [50], removed and allowed to cool. The contents of the tube were quantitatively transferred to 25 ml volumetric flask and volume was adjusted with HPLC grade water. Five milliliter of this solution was filtered through 0.45 µm Millipore membrane filter for derivatization. This protein content in glass vial was stored in freezer at −20°C.

#### Derivatization procedure

Aliquots of extract and working amino acid standard solution prepared in the previous section were concentrated and dried under vacuum (37°C, 20 mmHg). Then a coupling reagent (methanol/water/TEA, 2∶2∶1, v/v) was added and the solution was mixed and dried immediately under vacuum. After this, PITC reagent (methanol/TEA/water/PITC, 7∶1∶1∶1, v/v) was added and allowed to stand at room temperature for 20 min before drying in vacuum. PITC derivatives were dissolved in sodium acetate buffer (mobile phase A) (Table 2).

**Table 2:** Gradient program employed for the separation of PITC derivatized amino acids^a^.

Run time^b^ (min)	Flow rate (ml/min)	% Buffer A^c^	% Buffer B (60% acetonitrile in water)
0	1	100	0
0.1	1	95	5
5	1	90	10
14	1	90	10
25	1	60	40
30	1	50	50
35	1	40	60
40	1	10	90
52	1	10	90
62	1	95	5
65	1	100	0

^a^ Column temperature was maintained at 39°C.

^b^ Run time was 62 min with 3 min column regeneration time.

^c^ Sodium acetate buffer [19 g of sodium acetate trihydrate was dissolved in 1 l of HPLC grade water. To this 0.5 ml of TEA was added and the contents were mixed properly. The pH of the solution was adjusted to 6.4 with glacial acetic acid and filtered. To the filtrate (940 ml) acetonitrile (60 ml) was added, mixed and filtered through a 0.22 µm Millipore membrane].

#### Analytical chromatographic conditions

The injection volume of the sample and standard was 20 µl. Separation of amino acids was performed on reverse phase C-18 column (5 µm, 150×4.6 mm) (Pickering Laboratories, Inc., Mountain-View, California, USA), with sodium acetate buffer, pH 6.4 (mobile phase A) and ACN : H_2_O :: 6 : 4 (mobile phase B) with gradient mode of operation. The detector setting was: Gain = 5, Temperature = 39°C and Pressure = 250 kPa. The absorbance at 254 nm was recorded and used for calculation. The actual chromagraphic conditions in the present investigation have been depicted in Table 2.

### Fatty Acid Analysis

Fat and fatty acids were extracted from the sample by hydrolytic method. Pyrogallic acid was added to minimize oxidative degradation of fatty acids during analysis. The fat was extracted into ether, methylated to FAMEs using BF_3_ in methanol, 14% (w/w). FAMEs were quantitatively measured by GC. Total fat was calculated as sum of individual fatty acids. Saturated and monounsaturated fats were calculated as sum of respective fatty acids [51].

#### Extraction of fat from sample

Accurately ground and homogenized 1 g of phytococktail was weighed into a Mojonnier flask (Cole-Parmer, Mumbai, India). Then 100 mg of pyrogallic acid and a few boiling granules were added to the flask. After that, 2 ml ethanol was added and mixed well. The flask was placed in a shaking water bath at 80°C set at moderate agitation speed for 40 min. Then the contents of the flask were mixed in a vortex mixer for 10 min and cooled to room temperature. An adequate amount of ethanol was added to fill bottom reservoir of the flask and mixed gently. Diethyl ether (25 ml) was then added to the Mojonnier flask, sealed with stopper and placed in centrifuge basket. The basket was placed in wrist action shaker and shaken for 5 min. After that, 25 ml petroleum ether was added, sealed with stopper, shaken for 5 min and centrifuged at 600 rpm for 5 min. The ether (top) layer was decanted into 150 ml beaker. This extraction procedure was repeated thrice and the extracts were combined. Then the ether was evaporated slowly on steam bath, using nitrogen stream to aid evaporation. The remaining residue in the beaker contained extracted fat.

#### Fatty Acid Methyl Esters (FAMEs) preparation

Extracted fat residue was dissolved in 3 ml each of chloroform and diethyl ether. Then the mixture was transferred to glass vial and evaporated to dryness at 40°C in water bath under nitrogen stream. Then 2 ml BF_3_-methanol (14%, w/w) and 1 ml toluene were added and sealed. The vial was heated in oven at 100°C for 45 min with gentle shaking with 10 min interval and cooled to room temperature. After that 5 ml water, 1 ml hexane and 1 g Na_2_SO_4_ was added into the vial, closed and shaken for 1 min. The layers were allowed to separate and the top layer (containing FAMEs) was carefully transferred to another vial containing 1 g Na_2_SO_4_ and finally filtered through 0.22 µm Millipore membrane filter. The filtrate (extracted FAMEs) was then used for further GC-FID analysis.

#### GC-FID analysis

A mixture of 37 FAMEs standard solution of varied concentration of 2% and 4% as aforementioned was diluted with 10 times of hexane, filtered, and stored at –20°C for GC-FID analysis. FAMEs were identified by direct comparison with the standard mixture. The percentage of individual FAMEs was made in relation to total area of the chromatogram. As different FAMEs have similar carbon chain length, it was assumed that they have the same response factor and volatility, allowing a direct comparison of the peak areas to determine the sample composition.

A GC-4000A system (East & West Analytical Instruments, Beijing, China) configured with flame ionization detector, split/split-less mode injector (5 ml/min), column oven temperature programming sufficient to implement a hold-ramp-hold sequence, Hewlett Packard capillary column HP-88, 100 m×0.25 mm×0.20 µm film (Agilents Technologies Ltd., Santa Clara, CA, USA) was used in the present investigation. Data acquisition was performed on A5000 Chromatogram Data Processing Workstation (Workstation Software, Version 1.6 with an interface board).

GC-FID was employed for analysis of FAMEs. Injector port and FID detector temperature were programmed at 250°C and 280°C, respectively. The oven temperature was programmed as follows: 80°C hold 5 min; 80°C to 140°C @ 8°C/min (7.5 min) hold 10 min; 140°C to 220°C @ 3°C/min (26.5 min); and 220°C to 240°C @ 2°C/min (10 min) hold10 min. Nitrogen, hydrogen and zero air were used as the carrier gas, reaction gas and detector gas at pressure of 0.25 MPa, 0.05 MPa, and 0.020 Mpa respectively at a flow rate of 1 ml/min. The injection split ratio was 1∶50. The injection volume was 1 µl in the split/split-less injection mode.

### Determination of Mineral Contents

#### Mineralization of the samples

The hot block digestion method was employed for digestion of the sample with QBlock (Questron Technologies Corp., Mississauga, Germany) equipment. An amount of 0.5 g of the dry phytococktail was added in a polypropylene vial and placed inside a fume hood. After that, 10 ml HNO_3_ and 2 ml H_2_O_2_ were added and then allowed the sample to outgas before loading them on QBlock. The digestion program was set for digestion of food sample (Receipe: H-Food-1) and reagents were added according to the manufacturer’s instructions. It was then diluted to volume with deionized water and was stored in a clean polypropylene bottle.

#### Analysis of mineral elements

The mineral elements were determined using an Inductively Coupled Plasma Optical Emission Spectrometer (ICP-OES) (Varian, VISTA-MPX, CCD Simultaneous ICP-OES, United States) [52]. The plasma conditions were as follows: RF power 1000 W, nebulizer flow 0.5 l/min, auxiliary flow 1 l/min, plasma flow 15 l/min and sample flow 1.5 ml/min. The wavelength used in the instrument and method detection limits for elements *viz.* calcium, iron, sodium, potassium, magnesium, phosphorus, zinc, chromium, manganese, nickel, copper and cobalt were 317.93, 238.20, 589.59, 766.49, 279.55, 177.43, 213.86, 267.72, 257.61, 231.60, 327.40 and 238.89 nm and 0.8, 0.3, 0.2, 0.3, 0.05, 4, 0.2, 0.9, 0.5, 0.1, 1.5 and 0.7 µg/l, respectively.

### Determination of Nutritive Value

#### Determination of total carbohydrate

Carbohydrate content was estimated by anthrone method [53]. In brief, 100 mg of dried and powdered phytococktail was weighed and took into a boiling tube. Carbohydrates were hydrolysed by keeping in a boiling water bath for 3 h with 5 ml of 2.5 N HCl and cooled to room temperature. The resultant solution was then neutralized with Na_2_CO_3_ until the effervescence ceases. The volume was made up to 100 ml followed by centrifugation. The supernatant was collected and 0.5 ml and 1 ml aliquots were taken for analysis. Anthrone reagent was prepared freshly by dissolving 200 mg anthrone in 100 ml of ice cold 95% H_2_SO_4_. Stock solution of glucose standard was prepared by dissolving 100 mg glucose in 100 ml distilled water and then 10 ml of stock was diluted to 100 ml with distilled water to prepare the working standard. It was stored in refrigerator after adding a few drops of toluene. The standards were prepared by taking 0 (blank), 0.2, 0.4, 0.6, 0.8 and 1 ml of the working standard. The volume was made up to 1 ml in all tubes including the sample tubes by adding distilled water. Then 4 ml of anthrone reagent was added and heated for 8 min in a boiling water bath. It was then cooled rapidly and the green to dark green color was measured at 630 nm. A standard curve was drawn by plotting concentration of the standards on the *x-*axis versus absorbance on the *y-*axis. From the standard curve, the carbohydrate concentration in the sample tube was calculated as following:

Amount of carbohydrate present in 100 mg of the sample = (mg of glucose/volume of test sample)×100.

#### Determination of total protein

Protein content was estimated by Lowry’s method [54]. Dried and powdered phytococktail (25 mg) was heated for 1 h with 2 ml of 1 N NaOH and filtered. The volume was made up to 5 ml with distilled water, centrifuged and supernatant was used as a test sample. Then 100 µl of test solution was diluted with water to make 1 ml, followed by addition of 5 ml of Lowry’s reagent [prepared by mixing 2% Na_2_CO_3_ in 0.1 N NaOH, 1% NaK Tartrate in H_2_O and 0.5% CuSO_4_.5 H_2_O in H_2_O with a ratio (vol : vol) of 100 : 1 : 1], mixed and incubated for 10 min at room temperature. Then 0.5 ml of 1 N Folin’s phenol reagent was added and the solution was kept for 30 min at room temperature after mixing. Absorbance was measured at 660 nm. A standard curve was prepared by using bovine serum albumin (Fraction V) as standard. The concentration of protein in the sample was determined from the standard curve. Protein concentration was reported as mg/100 g of dry weight.

#### Determination of crude fat

Crude fat was determined with the method described by previous investigators [55]. Two grams moisture-free phytococktail powder was extracted with petroleum ether in a Soxhlet extractor at 40–50°C for 6–8 h. After boiling with petroleum ether, the residual solvent was filtered and the filtrate was evaporated in a pre-weighed beaker. Increase in the weight of beaker gave the crude fat and it was expressed in mg/100 g of phytococktail material.

#### Nutritive value

Nutritive value of the phytococktail was calculated based on the energy value available per kg of the macro nutrient. Normally, protein, carbohydrate and fat yield 4.0, 4.0 and 9.0 kcal of energy per gram respectively. Therefore the nutritive value (NV) was calculated as, [(4×% protein)+(4×% carbohydrate)+(9×% fat)] [56].

## Results

### Vitamin Content

#### RRLC-MS/MS determination of water- and fat-soluble vitamins

**Figure 1:**
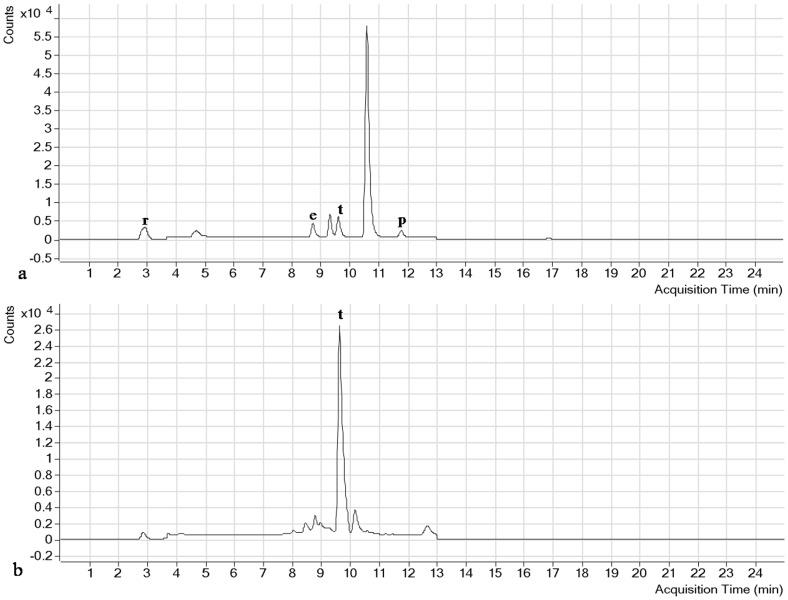
RRLC-MS/MS TIC chromatogram of a. Fat-soluble vitamin standards, b. Phytococktail sample under the optimum analysis conditions. **r:** retinol, **e:** ergocalciferol, **t:** tocopherol, **p:** phylloquinone.

**Figure 2:**
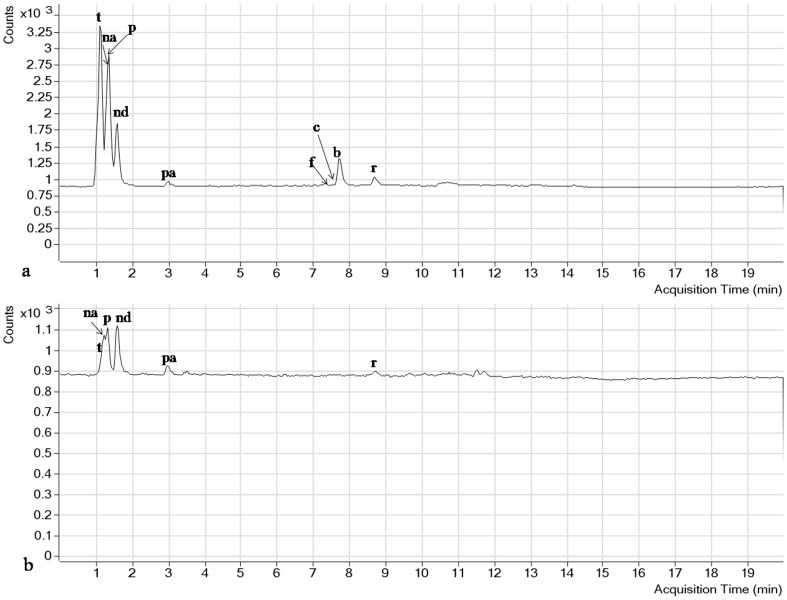
RRLC-MS/MS TIC chromatogram of a. Water-soluble vitamin standards, b. Phytococktail sample under the optimum analysis conditions. **t:** thiamine, **na:** nicotinic acid, **p:** pyridoxine, **nd:** nicotinamide, **pa:** pantothenic acid, **f:** folic acid, **c:** cyanocobalamin, **b:** biotin, **r:** riboflavin.

**Figure 3:**
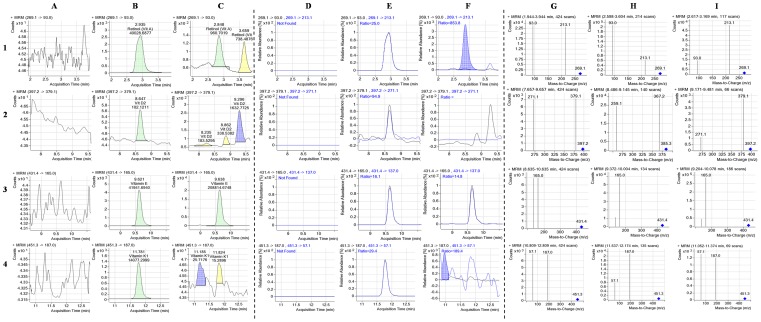
Extracted RRLC-MRM ion chromatogram of the analytes. The quantifier, qualifier and MRM transitions of extracted ions for blank, four fat-soluble standards and phytococktail sample have been shown for **1A–1I:** vit A, **2A–2I:** vit D_2_, **3A–3I:** vit E and **4A–4I:** vit K_1_.

**Figure 4:**
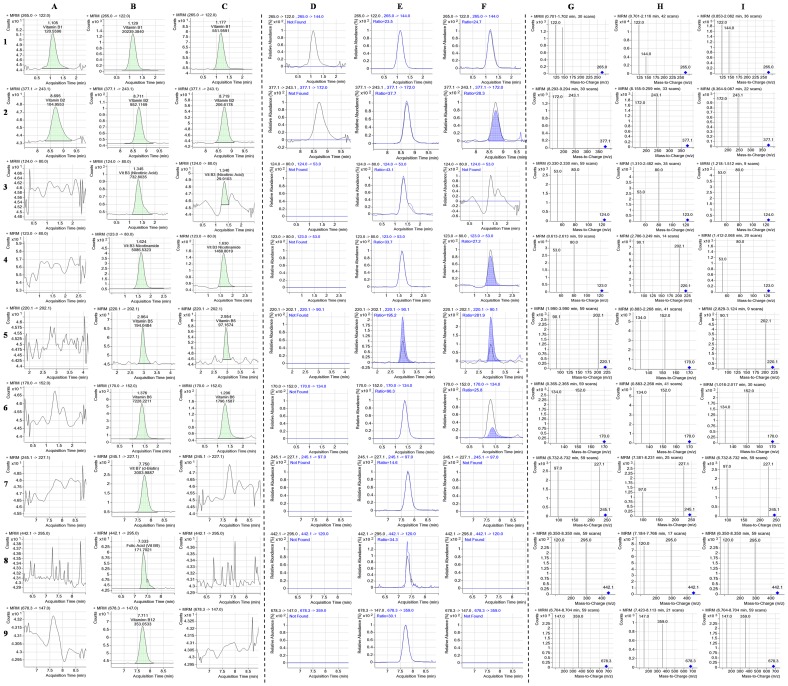
Extracted RRLC-MRM ion chromatogram of the analytes. The quantifier, qualifier and MRM transitions of extracted ions for blank, nine water-soluble standards and phytococktail sample have been shown for **1A–1I:** vit B_1_, **2A–2I:** vit B_2_, **3A–3I:** vit B_3_ (nicotinic acid), **4A–4I:** vit B_3_ (nicotinamide), **5A–5I:** vit B_5_, **6A–6I:** vit B_6_, **7A–7I:** B_7_, **8A–8I:** vit B_9_ and **9A–9I:** vit B_12_.

Analysis of water-soluble vitamins depends largely on the selection of a suitable chromatographic column for separation with enhanced resolution. The smaller system volume, high throughput, smaller particle-size and high pressure in RRLC column gives the advantage of separating more peaks in shorter run time. A number of columns such as Nova-Pack C18 (150×3.9 mm, 4 µm particle size), Separon C18 (150×3 mm, 7 µm particle size) and MetaChem Polaris C18-A (150×4.6 mm, 3 µm) were used to separate the water and fat-soluble vitamins with HPLC [6], [57], [58]. In the present study, the performance of two different reversed phase (RP) columns was tested in the optimization process for the separation of the 9 water-soluble vitamins and 4 fat-soluble vitamins studied. A short C18 column (2.1×50 mm, 1.9 µm particle diameter) and a longer C18 column (2.1×100 mm, 2.7 µm particle size) of Agilent Poroshell 120 EC-C18 were compared for analysis. The longer column was found to have a much better separation among the analytes, including B_1_, B_3_ and B_3_ that were weakly retained in the C18 stationary phase along with short duration of analysis (9 min) and D_2_ that had very close retention time. In our study, the selection of column was in agreement with previous reports in which the long C18 column with small particle size was found to produce optimized peak separation [6], [59]. The complete chromatograms of the 9 water-soluble and 4 fat-soluble vitamins and the detailed compound chromatograms describing the quantifier, qualifier and MRM transitions of blank, standard and all individual vitamins are shown in Fig. 1, Fig. 2, Fig. 3 and Fig. 4. The separation of all the studied compounds was successfully achieved.

After achieving optimum separation, the optimization of different MS/MS detection parameters was carried out to confirm the structural identities of analytes and instrumental sensitivity. It is critical to optimize the quantitative ion and impact energy for determination of multiple vitamins by MRM. Positive ESI ionization modes were selected for the production of typical precursor and product ions of each compound with the direct infusion of standard solutions. Selection of precursor ions was based on the most abundant mass-to-charge (m/z) values and subsequently, two product ions (qualifier ion and quantifier ion) for each precursor were selected. Quantification was achieved using the most intense product ion while the other was employed for authentication of compound identity. The precursor and product ions chosen for each studied compound, the collision energies, fragmentor voltage and dwell time values employed for their detection have been summarized in Table 3. All vitamins were detected as [M+H]^+^ except cyanocobalamin where the doubly charged ion [M+H]^2+^ at m/z 678.3 was selected as the parent ion. Thiamine (B_1_) and pyridoxine (B_6_) were observed as the loss of associated chloride, whereas calcium pantothenate was determined as pantothenic acid (B_5_) by the loss of calcium. Ergocalciferol (D_2_) and phylloquinone (K_1_) lost one molecule of water and phytyl chain respectively to produce the product ions in the collision-induced decomposition (CID) while fragmentation of D-α-tocopherol (E) induced the cleavage of the chromanol ring that led to the production of product ions. All other parameters in the ESI detection of the studied water- and fat-soluble vitamins, *viz.* capillary temperature, drying gas flow, nebulizer pressure, capillary voltage, delta EMV, resolution and scanning mode (MRM) were optimized and the values that offered the most excellent response for all vitamins have been depicted in Table 3.

**Table 3:** Optimized MS/MS transition parameters.

Analytes	Precursor ion(m/z)	MRM Transition		Fragmentor voltage (V)	Dwell time (ms)
		Quantifier ion (m/z) (collisionenergy, eV/V)	Qualifier ion (m/z) (collisionenergy, eV/V)		
Fat-soluble vitamins					
Retinol (A)	269.1 [M+H]^+^	269.1 → 93.0 (20)	269.1 → 213.1 (13)	100	100
Ergocalciferol (D_2_)	397.2 [M+H]^+^	397.2 → 379.1 (10)	397.2 →271.1 (11)	120	50
D-α-Tocopherol (E)	431.4 [M+H]^+^	431.4 → 165.0 (18)	431.4 → 137.0 (45)	120	50
Phylloquinone (K_1_)	451.3 [M+H]^+^	451.3 → 187.0 (23)	451.3 → 57.1 (36)	140	50
Water-soluble vitamins					
Thiamine (B_1_)	265.0 [M+H]^+^	265.0 → 122.0 (10)	265.0 → 144.0 (8)	80	100
Riboflavin (B_2_)	377.1 [M+H]^+^	377.1 → 243.1 (22)	377.1 → 172.0 (41)	140	100
Nicotinic acid (B_3_)	124.0 [M+H]^+^	124.0 → 80.0 (10)	124.0 → 53.0 (8)	80	100
Nicotinamide (B_3_)	123.0 [M+H]^+^	123.0 → 80.0 (20)	123.0 → 53.0 (32)	100	100
D-Pantothenic acid (B_5_)	220.1 [M+H]^+^	220.1 → 90.1 (9)	220.1 → 202.1 (6)	70	100
Pyridoxine (B_6_)	170.0 [M+H]^+^	170.0 → 134.0 (19)	170.0 → 152.0 (8)	80	100
D-Biotin (B_7_)	245.1 [M+H]^+^	245.1 → 227.1 (9)	245.1 → 97.0 (30)	70	100
Folic acid (B_9_)	442.1 [M+H]^+^	442.1 → 295.0 (15)	442.1 → 120.0 (40)	60	100
Cyanocobalamin (B_12_)	678.3 [M+H]^2+^	678.3 → 147.0 (41)	678.3 → 359.0 (20)	140	100

#### Validation results

After selection and optimization of the RRLC separation conditions and MS/MS detection parameters, evaluation of instrumental intra-day and inter-day precision was carried out. Intra-day precision was evaluated through the successive injection (n = 5) of standards that were injected consecutively in the same day and repeated in four different days for assessment of inter-day precision (n = 20). The obtained results of intra- and inter-day precision (% RSD values) for retention time and recoveries have been depicted in Table 4. The intra- and inter-day precisions of responses are illustrated in Table 5. For water-soluble vitamins, the intra- and inter-day RSD values of retention time ranged between 0.024–0.172% and 0.085–0.201% respectively. The intra- and inter-day RSD values of retention time for fat-soluble vitamins ranged between 0.009–0.85% and 0.022–0.152% respectively. For water-soluble vitamins, the recovery precision for the same day ranged between 0.17–0.29%, 0.22–0.27%, and 0.22–0.29% for recoveries of three different concentrations of standards *viz.* µgml^−0.1^, µgml^−0.5^ and µgml^−1^ respectively. The RSD values for inter-day were slightly higher and ranged between 0.39–0.52%, 0.40–0.47% and 0.32–0.44%. The mean recoveries ranged between 88.95–107.07%, 98.35–103.27% and 98.77–102.33%. The intra-day precision of recovery for the fat-soluble vitamins ranged between 0.16–0.37%, 0.18–0.28% and 0.19–0.29%. The inter-day RSDs were somewhat higher and ranged between 0.43–0.57%, 0.31–0.44% and 0.30–0.37%. The mean recoveries for fat-soluble vitamins ranged between 97.60–104.14%, 97.67–103.55% and 97.71–100.75% (Table 6). The intra- and inter-day precisions for recoveries and retention time were always below 0.6% and 0.3%.

**Table 4:** Intra- and inter-day precision of retention time, recovery and linearity (n = 5).

Analytes	RT precision (% RSD)		Recovery precision (% RSD) and mean recovery (%)									Linearity range (ppb)
	Intra-day	Inter-day	Mean recovery	Intra-day	Inter-day	Mean recovery	Intra-day	Inter-day	Meanrecovery	Intra-day	Inter-day	
Fat-soluble vitamins			100 ppb			500 ppb			1000 ppb			
Retinol (A)	0.054	0.146	99.16	0.28	0.54	103.55	0.28	0.43	99.72	0.22	0.31	100–1000
Ergocalciferol (D_2_)	0.085	0.152	97.60	0.16	0.43	100.30	0.20	0.31	99.30	0.23	0.35	100–1000
D-α-Tocopherol (E)	0.033	0.134	103.36	0.20	0.46	97.67	0.19	0.44	97.71	0.19	0.37	100–1000
Phylloquinone (K_1_)	0.009	0.022	101.91	0.37	0.46	97.74	0.18	0.35	97.79	0.29	0.35	100–1000
Water-soluble vitamins			10 ppb			50 ppb			100 ppb			
Thiamine (B_1_)	0.137	0.140	107.07	0.17	0.41	98.74	0.22	0.41	98.77	0.29	0.40	10–100
Riboflavin (B_2_)	0.024	0.085	90.93	0.25	0.39	103.27	0.23	0.47	101.56	0.25	0.41	10–100
Nicotinic acid (B_3_)	0.172	0.196	88.95	0.29	0.52	100.63	0.27	0.42	100.30	0.28	0.42	10–100
Nicotinamide (B_3_)	0.078	0.125	90.58	0.26	0.45	101.62	0.26	0.45	101.68	0.26	0.36	10–100
D-Pantothenic acid (B_5_)	0.139	0.201	92.24	0.23	0.41	99.41	0.24	0.40	99.38	0.29	0.44	10–100
Pyridoxine (B_6_)	0.141	0.153	91.80	0.25	0.48	99.71	0.24	0.41	99.61	0.25	0.44	10–100
D-Biotin (B_7_)	0.041	0.110	99.23	0.23	0.48	98.35	0.23	0.42	98.77	0.28	0.32	10–100
Folic acid (B_9_)	0.049	0.092	95.81	0.24	0.52	102.88	0.24	0.43	102.33	0.29	0.32	10–100
Cyanocobalamin (B_12_)	0.162	0.192	89.53	0.25	0.51	99.71	0.23	0.40	99.84	0.22	0.41	10–100

**Table 5:** Intra- and inter-day precision of response.

Analytes	Precision of response					
	Intra-day	Inter-day	Intra-day	Inter-day	Intra-day	Inter-day
Fat-soluble vitamins	100 ppb		500 ppb		1000 ppb	
Retinol (A)	0.05	0.07	0.01	0.03	0.03	0.04
Ergocalciferol (D_2_)	5.02	8.70	3.41	5.04	2.14	3.20
D-α-Tocopherol (E)	0.05	0.07	0.01	0.02	0.04	0.05
Phylloquinone (K_1_)	0.16	0.22	0.04	0.06	0.12	0.15
Water-soluble vitamins	10 ppb		50 ppb		100 ppb	
Thiamine (B_1_)	0.13	0.18	0.03	0.04	0.06	0.08
Riboflavin (B_2_)	0.82	1.06	0.32	0.43	0.93	1.56
Nicotinic acid (B_3_)	1.44	2.04	0.57	0.69	0.69	1.02
Nicotinamide (B_3_)	0.20	0.41	0.07	0.09	0.15	0.23
D-Pantothenic acid (B_5_)	7.26	8.52	2.21	2.85	1.86	3.22
Pyridoxine (B_6_)	0.13	0.23	0.06	0.07	0.12	0.20
D-Biotin (B_7_)	0.44	0.69	0.16	0.21	0.25	0.36
Folic acid (B_9_)	7.68	8.33	2.24	2.68	2.02	3.22
Cyanocobalamin (B_12_)	2.32	3.27	1.04	1.39	1.76	2.28

**Table 6:** Validation data for determination of fat- and water-soluble vitamins (n = 3).

Analyte	RT (min)^a^	RT RSD (%)^b^	Regression equation	*R^2^* c	LOD (ppb)^d^	LOQ (ppb)^e^
Fat-soluble vitamins						
Retinol (A)	2.9387±0.0091	0.3088	y = 40.0574x + 339.2849	0.9991	0.18	0.61
Ergocalciferol (D_2_)	8.6523±0.0225	0.2598	y = 0.0923x + 11.8327	0.9980	47.82	159.41
D-α-Tocopherol (E)	9.6253±0.0131	0.1356	y = 41.8311x − 139.1714	0.9996	0.18	0.59
Phylloquinone (K_1_)	11.7927±0.0220	0.1862	y = 14.0609x − 412.1498	0.9911	0.41	1.37
Water-soluble vitamins						
Thiamine (B_1_)	1.1310±0.0017	0.1531	y = 201.1694x − 429.8190	0.9969	0.04	0.13
Riboflavin (B_2_)	8.7147±0.0035	0.0403	y = 8.2510x + 175.5517	0.9861	0.86	2.87
Nicotinic acid (B_3_)	1.3417±0.0067	0.4963	y = 7.4249x + 57.4607	0.9678	0.86	2.88
Nicotinamide (B_3_)	1.6220±0.0017	0.1068	y = 51.5908x + 315.5337	0.9775	0.14	0.46
D-Pantothenic acid (B_5_)	2.9730±0.0276	0.9291	y = 1.9568x + 5.3674	0.9948	2.85	9.51
Pyridoxine (B_6_)	1.3747±0.0023	0.1680	y = 71.7554x + 399.2698	0.9906	0.08	0.27
D-Biotin (B_7_)	7.7440±0.0060	0.0775	y = 30.9773x + 73.1416	0.9883	0.20	0.66
Folic acid (B_9_)	7.3167±0.0225	0.3075	y = 1.6740x + 6.3379	0.9994	3.30	11.00
Cyanocobalamin (B_12_)	7.7023±0.0168	0.2178	y = 3.6046x + 22.2344	0.9733	1.27	4.22

^a^ Retention time (RT) in min.

^b^ Relative standard deviation (RSD).

^c^ Regression coefficients (*R^2^*).

^d^ Limits of detection (LOD, 3.S/N).

^e^ Limits of quantitation (LOQ, 10.S/N).

The linearity was also examined through the calibration curves that were obtained by plotting concentration against peak area. A series of three concentration points as described earlier was prepared and each solution was injected ten times for each analyte. The linearity was excellent (*R* ^2^>0.99) for most of the water-soluble vitamins, except nicotinic acid, nicotinamide and cyanocobalamin (*R* ^2^ = 0.97−0.98), while, for all the fat-soluble vitamins the linearity was found to be very good (*R* ^2^>0.99) within the selected range of concentrations. We followed the U.S. Food and Drug Administration (USFDA), 2001 criterion where it was recommended that the analyte response at LOD should be consistently distinguished from the background noise and for this reason gradual dilution of the sample solution was carried out to determine the LOD and LOQ at signal-to-noise (S/N) ratio 3 and 10 respectively. LODs and LOQs have been depicted in Table 6 as mean of three replicates.

**Figure 5:**
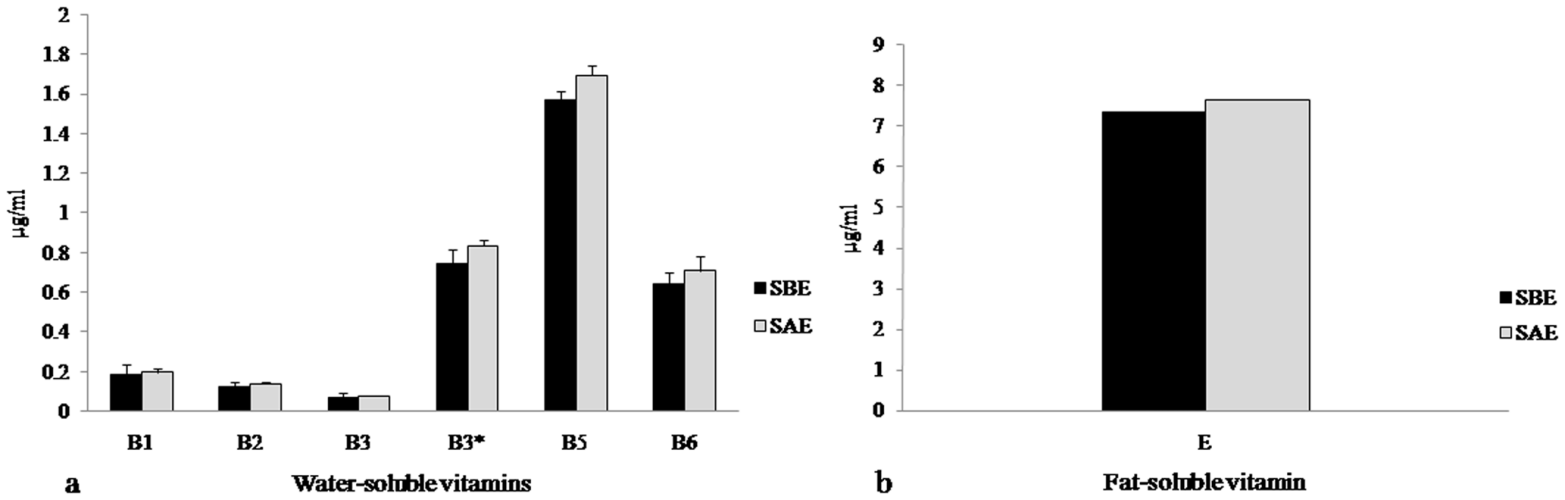
Recoveries of a. water- and b. fat-soluble vitamins (%) obtained from phytococktail spiked before extraction (SBE) and spiked after extraction (SAE).

**Figure 6:**
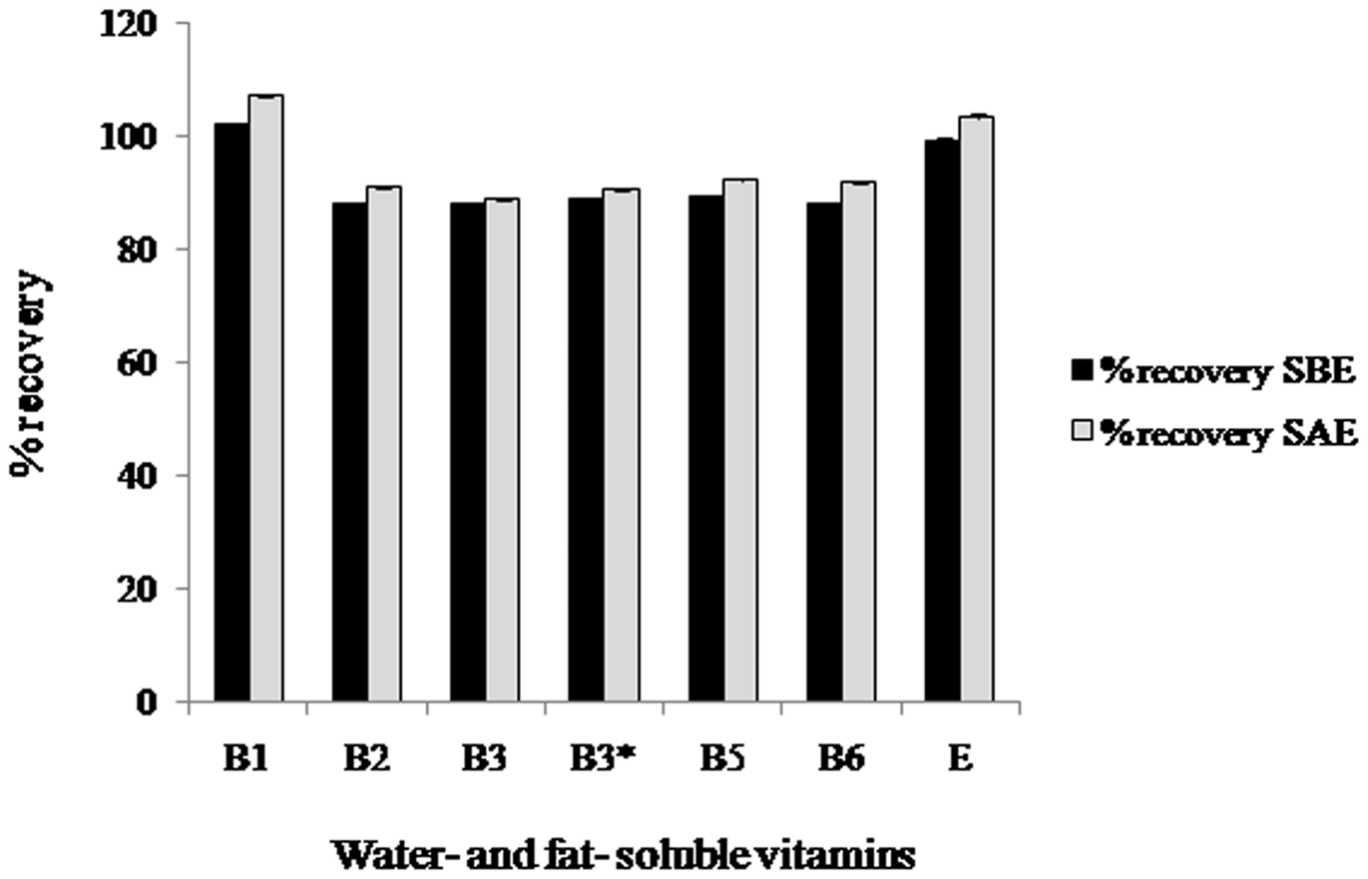
Mean content of water- and fat-soluble vitamins in phytococktail sample before extraction (SBE) and spiked after extraction (SAE).

We performed a recovery study to evaluate the accuracy of the complete method. Samples were spiked before and after the optimized extraction procedure. We have compared the values obtained from the spiked-recovery study and no significant matrix effect was observed during the extraction procedure (Fig. 5). The recoveries ranged between 87% and 107% that was considered to be suitable for simultaneous determination of heterogeneous compounds in hyphenated techniques (Fig. 6).

The results obtained from the analyses have been summarized in Table 7 and expressed as µg of vitamin per kg fresh weight (fw) of phytococktail.

**Table 7:** Content of fat- and water-soluble vitamins in phytococktail (n = 5).

Analytes	Content in phytococktail (µg/Kg)
Fat-soluble vitamins	
Retinol (A)	ND
Ergocalciferol (D_2_)	ND
D-α-Tocopherol (E)	4926.744±135.222
Phylloquinone (K_1_)	ND
Water-soluble vitamins	
Thiamine (B_1_)	121.620±9.345
Riboflavin (B_2_)	93.828±5.827
Nicotinic acid (B_3_)	53.910±5.332
Nicotinamide (B_3_)	557.072±17.161
D-Pantothenic acid (B_5_)	1169.080±59.531
Pyridoxine (B_6_)	485.132±12.973
D-Biotin (B_7_)	ND
Folic acid (B_9_)	ND
Cyanocobalamin (B_12_)	ND

ND: Not detectable or<LOD.

### Amino Acid Composition

**Figure 7:**
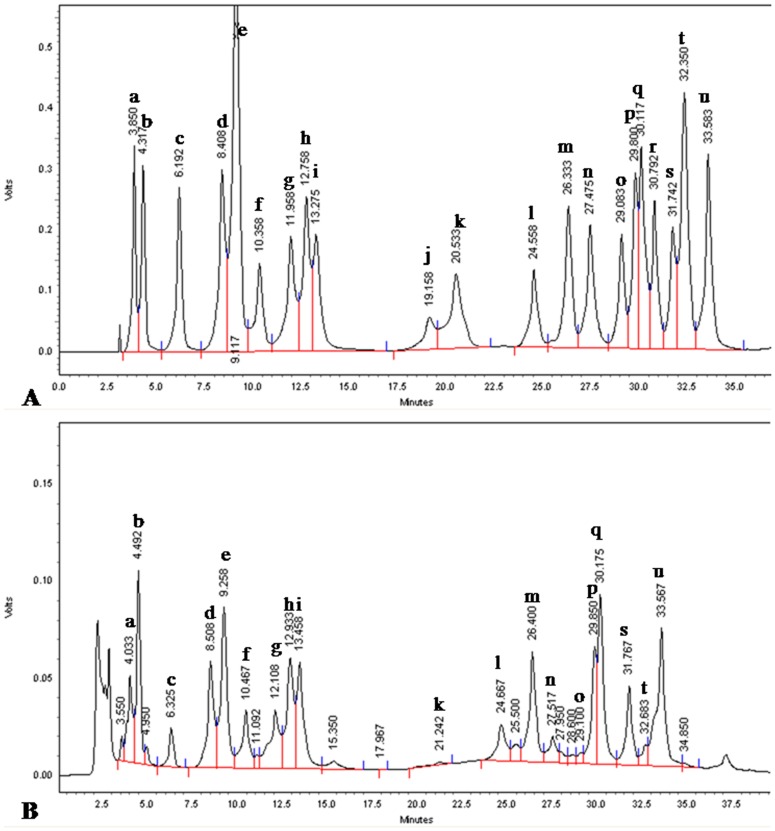
RP-HPLC chromatogram of A. 21 amino acid standards, B. Amino acid profile of the phytococktail. **a:** L-Arginine; **b:** L-Aspartic Acid; **c:** L-Glutamic Acid; **d:** L-Serine; **e:** Glycine; **f:** L-Histidine; **g:** L-Threonine; **h:** L-Alanine; **i:** L-Proline; **j:** L-2-amino-n-butyric acid; **k:** L-Valine; **l:** L-Methionine; **m:** L-Cystine HCl; **n:** L-Cystine; **o:** L-Isoleucine; **p:** L-Leucine; **q:** L-Nor Leucine; **r:** L-Tryptophan; **s:** L-Phenylalanine; **t:** L-Ornithine; **u:** L-Lysine.

The content of all amino acids studied using RP-HPLC with pre-column derivatization from the phytococktail has been summarized in Table 8. The sample peaks were compared with those of reference compounds analyzed under the same conditions (Fig. 7). The peaks were identified on the basis of comparison between the retention time of the standards of the amino acids and those in the sample. Quantitation was achieved through the external standard method using calibration curves fitted by linear regression analysis (Statistica 5.1, StatSoft, Tulsa, OK).

**Table 8:** Content, type of amino acid, retention time (RT), peak area as quantitated by RP-HPLC.

Amino acid	Abbreviation	Type	RT (min)	Peak area	Content (µg/g)
L-Arginine	Arg	Non essential	4.033	840667	933.667±10.066
L-Aspartic Acid	Asp	Non essential	4.492	1477469	1635.667±9.609
L-Glutamic Acid	Glu	Non essential	6.325	356571	355.667±11.015
L-Serine	Ser	Non essential	8.508	1352648	948.667±7.371
Glycine	Gly	Non essential	9.258	2067729	1046.333±11.015
L-Histidine	His	Essential	10.467	875468	1052.667±12.423
L-Threonine	Thr	Essential	12.108	1177030	958.333±13.650
L-Alanine	Ala	Non essential	12.933	1576317	1128.333±15.503
L-Proline	Pro	Non essential	13.458	1549047	1448.333±10.017
L-2-amino-n-butyric acid	Abu	Non essential	ND	ND	ND
L-Valine	Val	Essential	21.242	83423	76.333±7.638
L-Methionine	Met	Essential	24.667	639545	9485.667±10.017
L-Cystine HCl	Cys HCl	Non essential	26.400	1573488	1262.667±11.719
L-Cystine	Cys	Non essential	27.517	419992	369.667±11.930
L-Isoleucine	Ile	Essential	29.100	116971	133.333±10.599
L-Leucine	Leu	Essential	29.850	1218013	1051.667±10.693
L-Nor Leucine	Nor Leu	Non essential	30.175	1929186	1265.333±8.737
L-Tryptophan	Trp	Essential	ND	ND	ND
L-Phenylalanine	Phe	Essential	31.767	1006590	1157.333±9.504
L-Ornithine	Orn	Non essential	32.683	236800	115.333±18.771
L-Lysine	Lys	Essential	33.567	2260476	1334.333±9.609

ND: Not detectable.

**Figure 8:**
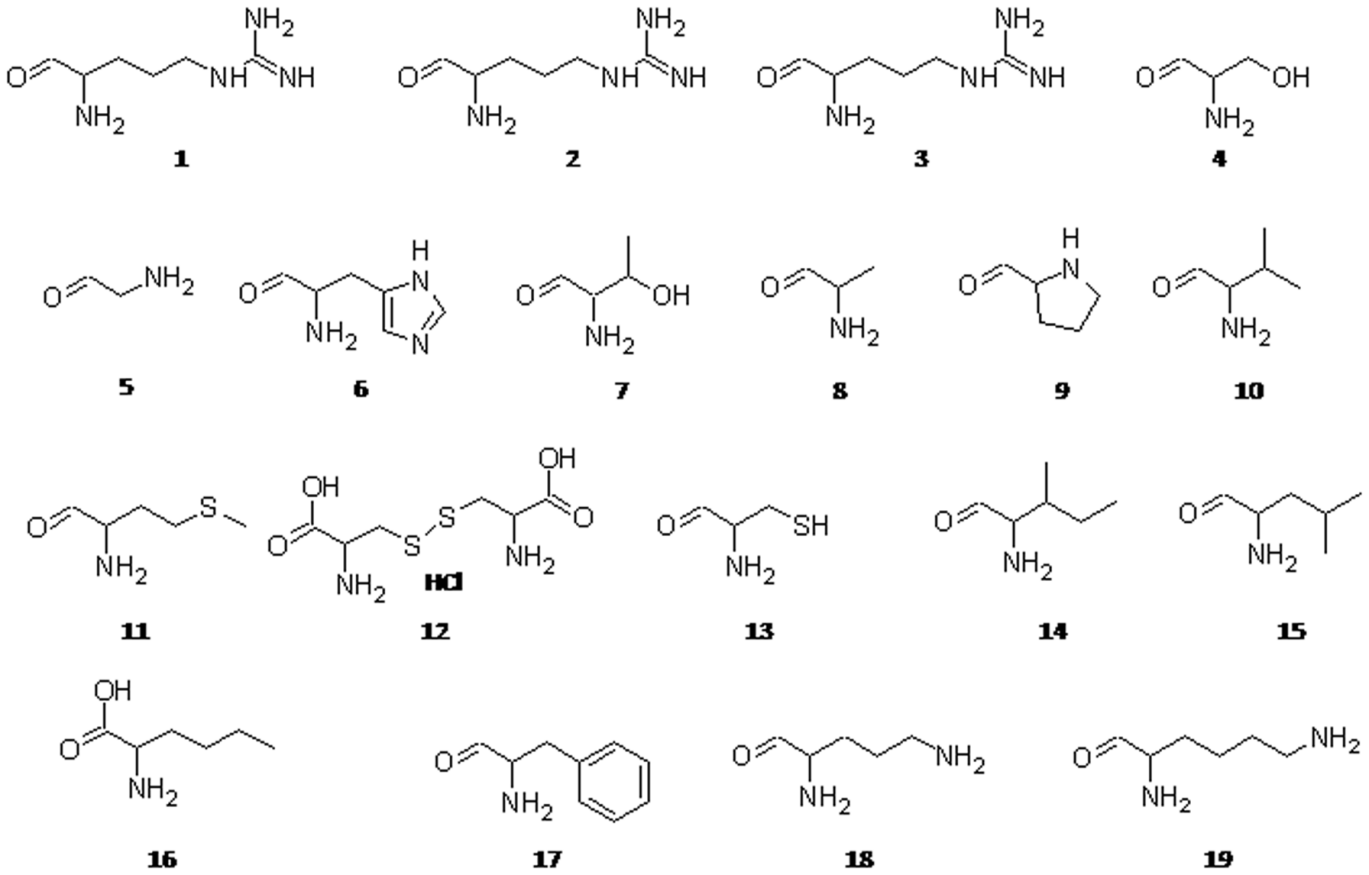
Different amino acids identified in the phytococktail by RP-HPLC. **1:** L-Arginine; **2:** L-Aspartic Acid; **3:** L-Glutamic Acid; **4:** L-Serine; **5:** Glycine; **6:** L-Histidine; **7:** L-Threonine; **8:** L-Alanine; **9:** L-Proline; **10:** L-Valine; **11:** L-Methionine; **12:** L-Cystine HCl; **13:** L-Cystine; **14:** L-Isoleucine; **15:** L-Leucine; **16:** L-Nor Leucine; **17:** L-Phenylalanine; **18:** L-Ornithine; **19:** L-Lysine.

The quantification of amino acids in the phytococktail revealed the amino acid profile with contribution of 19 amino acids including 8 essential and 11 non essential amino acids (Table 8, Fig. 8). The content of 19 amino acids quantified by RP-HPLC ranged between 70 and 9400 µg/g. The highest content was of L-Met (9485.67 µg/g) followed by L-Asp (1635.67 µg/g), L-Pro (1448.33 µg/g), L-Lys (1334.33 µg/g), L-nor Leu (1265.33 µg/g), L-Cys HCl (1262.67 µg/g) and L-Phe (1157.33 µg/g). L-Met was found to contribute to nearly 38% of the total amino acid content. Among the essential amino acids, L-methionine, L-phenylalanine, L-lysine, L-leucine and L-histidine were found to be the dominant contributors, while L-aspartic acid, L-proline, L-cystine HCl, L-nor leucine, L-alanine and glycine were found to be the major non essential amino acids in the phytococktail.

### Fatty Acid Profile

**Figure 9:**
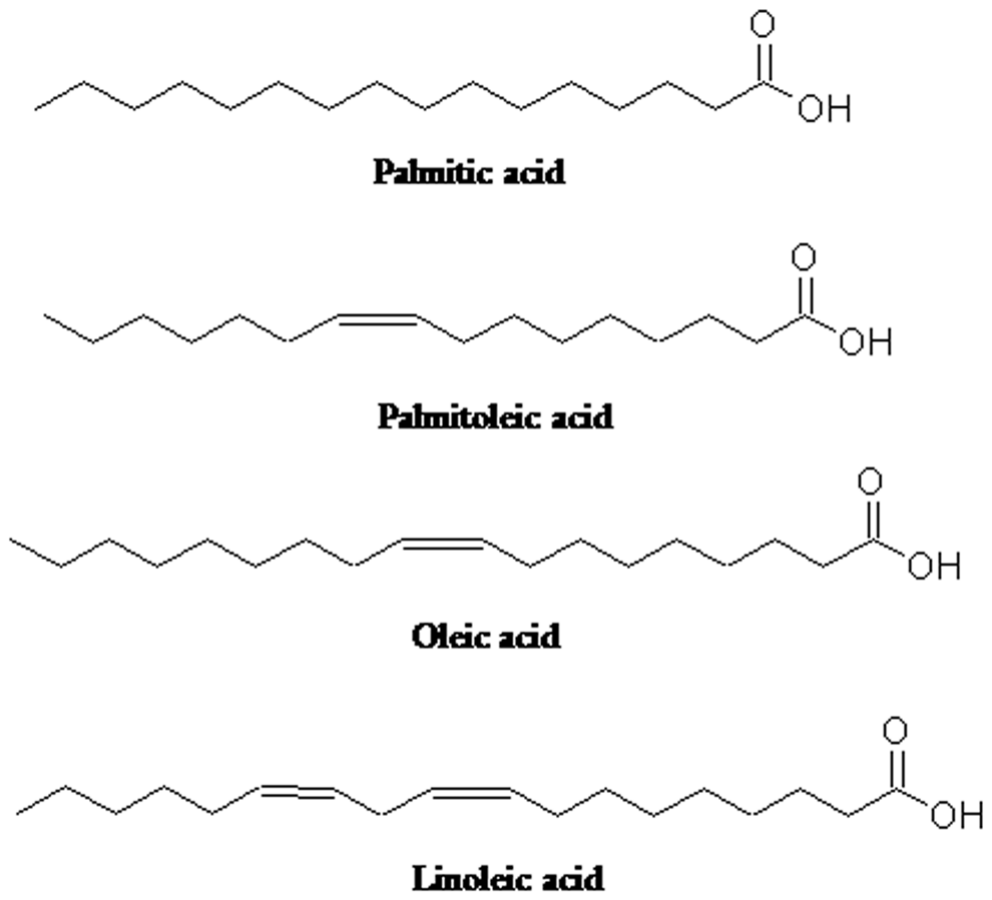
Different fatty acids identified in the phytococktail by GC-FID.

**Figure 10:**
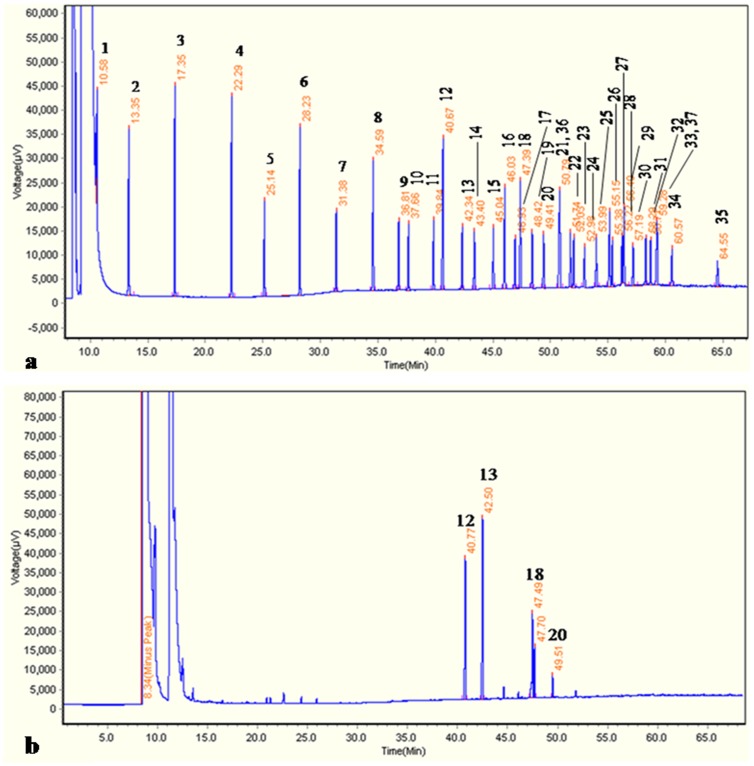
GC-FID chromatogram of a. 37 FAMEs standards, b. FAMEs of the phytococktail. **1:** Butyric acid methyl ester (C4∶0); **2:** Caproic acid methyl ester (C6∶0); **3:** Caprylic acid methyl ester (C8∶0); **4:** Capric acid methyl ester (C10∶0); **5:** Undecanoic acid methyl ester (C11∶0); **6:** Lauric acid methyl ester (C12∶0); **7:** Tridecanoic acid methyl ester (C13∶0); **8:** Myristic acid methyl ester (C14∶0); **9:** Myristoleic acid methyl ester (C14∶1); **10:** Pentadecanoic acid methyl ester (C15∶0); **11:** *cis*-10-Pentadecenoic acid methyl ester (C15∶1); **12:** Palmitic acid methyl ester (C16∶0); **13:** Palmitoleic acid methyl ester (C16∶1); **14:** Heptadecanoic acid methyl ester (C17∶0); **15:** *cis*-10-Heptadecenoic acid methyl ester (C17∶1); **16:** Stearic acid methyl ester (C18∶0); **17:** Elaidic acid methyl ester (C18∶1n9t); **18:** Oleic acid methyl ester (C18∶1n9c); **19:** Linolelaidic acid methyl ester (C18∶2n6t); **20:** Linoleic acid methyl ester (C18∶2n6c); **21:** Arachidic acid methyl ester (C20∶0); **22:** *cis*-11-Eicosenoic acid methyl ester (C20∶1); **23:** α-Linolenic acid methyl ester (C18∶3n3); **24:** Heneicosanoic acid methyl ester (C21∶0); **25:** *cis*-11,14-Eicosadienoic acid methyl ester (C20∶2); **26:** Behenic acid methyl ester (C22∶0); **27:** *cis*-8,11,14-Eicosatrienoic acid methyl ester (C20∶3n6); **28:** Erucic acid methyl ester (C22∶1n9); **29:** *cis*-11,14,17-Eicosatrienoic acid methyl ester (C20∶3n3); **30:** Arachidonic acid methyl ester (C20∶4n6); **31:** Tricosanoic acid methyl ester (C23∶0); **32:** *cis*-13,16-Docosadienoic acid methyl ester (C22∶2); **33:** Lignoceric acid methyl ester (C24∶0); **34:** Nervonic acid methyl ester (C24∶1); **35:** *cis*-4,7,10,13,16,19-Docosahexaenoic acid methyl ester (C22∶6n3); **36:** γ-Linolenic acid methyl ester (C18∶3n6); **37:** *cis*-5,8,11,14,17-Eicosapentaenoic acid methyl ester (C20∶5n3).

The analysis of fatty acid obtained from phytococktail revealed the presence of 4 major fatty acids (Table 9, Fig. 9) contributing to the total lipid content. The GC-FID chromatogram of the 37 FAMEs standards and the phytococktail sample were shown in Fig. 10. Palmitic acid was found to be the rich source of SFA and constituted ∼31% of the total lipid (Table 9). Among the UFAs, palmitoleic acid (43.47%), oleic acid (20.89%) and linoleic acid (4.31%) were prominent. MUFAs and PUFAs were 64.36% and 4.31% respectively of the total lipid content in the phytococktail.

**Table 9:** Fatty acid composition of the phytococktail.

Type of fatty acid	Name	CASNumber	Retentiontime (min)	Peakarea	Peakheight	Peakwidth	% Content intotal lipid	Content in phytococktail (mg/g)
Saturated fatty acid (SFA)								
Palmitic acid (C16∶0)	(9*Z*)-Hexadecanoic acid	57-10-3	40.77	187793	35964	0.486	31.33	36
Unsaturated fatty acid (UFA)								
Monounsaturated fatty acid (MUFA)								
Palmitoleic acid (C16∶1)	Hexadec-9-enoic acid	1120-25-8	42.50	260604	46199	0.458	43.47	50
Oleic acid (C18∶1n9c)	(9*Z*)-Octadec-9-enoic acid	112-80-1	47.49	125252	21364	0.418	20.89	24
Poly unsaturated fatty acid (PUFA)								
Linoleic acid (C18∶2n6c)	*cis,cis*-9,12-Octadecadienoicacid	60-33-3	49.52	25816	4661	0.244	4.31	4.9
∑ SFA							31.33	
∑ UFA							68.67	
∑ MUFA							64.36	
∑ PUFA							4.31	

The nutritional value of fat is determined by the PUFA/SFA ratio. Low MUFA/SFA ratio, low PUFA/SFA ratio, high PUFA/MUFA ratio and PUFA+MUFA/SFA ratio <2 is reported to maintain of low plasma and liver lipid concentration [60]. In our present investigation, the value of MUFA/SFA was 2.05, PUFA/SFA was 0.14, PUFA/MUFA was 0.07 and PUFA+MUFA/SFA was 2.19 as the MUFA content was very high in the phytococktail.

### Nutritive Value and Mineral Content

The nutritional profiling of the phytococktail revealed predominance of carbohydrate content followed by fat and protein content. The concentration of total carbohydrate, protein and fat of the phytococktail has been mentioned in Table 10. The nutritive value was calculated to be 114.71. Mineral composition of phytococktail has been depicted in Table 10.

**Table 10:** Major nutritional composition and mineral content of the phytococktail.

Nutritional composition	Content (mg/g)
Total carbohydrate	572.4
Total protein	30.4
Total fat	88.5
**Minerals**	**Content (mg/kg)**
Calcium	3850.3
Iron	428.2
Sodium	41398.1
Potassium	7046.8
Magnesium	853.5
Phosphorus	973.3
Zinc	19.7
Chromium	6.7
Manganese	25.9
Nickel	0.5
Copper	6.7

## Discussion

The therapeutic efficacy of bioactive compounds from plant sources has got great interest in pharmacognosy, pharmacology and natural product research. The efficacy and safety of treatment of diseases with herbal interventions has been in vogue since ages. The therapeutic actions of botanical drugs are well documented in traditional system of medicine in India, China, Africa, Europe and other parts of the world [61]. In addition, precise identification of edible plants and cataloging of food products and nutraceuticals have become important issues in agricultural and food sciences. Considering the prevalence of oxidative stress and nutritional deficiency at high altitude, we aimed at developing a phytococktail that could not only possess high nutritional attributes but also have antioxidant properties. Analyses of nutrients in the phytococktail comprising of sea buckthorn (*Hippophae rhamnoides*), apricot (*Prunus armeniaca*) and roseroot (*Rhodiola imbricata*) formulated in the present study revealed that carbohydrate, protein, fat, vitamins and minerals were present in good quantity with high nutritive value.

Estimation of free forms of fat- and water-soluble vitamins in different samples can be achieved by two different approaches. The first is to separate the entire vitamin forms in a single analysis, while the second is to optimize two individual methods for a superior and precise measurement of the diverse vitamins [59]. In the present study, we have developed two different sample preparation and chromatographic methods that share the same column type and elution solvents, employed for two methods having different gradients for the analysis of both types of vitamins in the phytococktail which could prove effectiveness in providing an accurate quantification of the free vitamin forms. The selection of the mobile phase and the composition of organic solvents are extremely critical in RRLC separation. The highly polar water-soluble vitamins have poor retention on RP columns and thus presence of ion pair reagents such as acetic acid, trifluoroacetic acid, pentafluoropropionic acid, formic acid or heptafluorobutyric acid in the mobile phase has been shown to improve the separation and retention of these compounds. Of these reagents, formic acid is a convenient, contamination-free alternative for preparing elution solvents for RRLC separations and after comparing these reagents, formic acid was found to be appropriate. However, the ion pair reagents often produce high background levels inside the mass spectrometer and to neutralize this negative effect we have developed a rapid and sensitive method using ammonium formate in the mobile phase to retain hydrophilic components. After taking lead from the previous reports [6], [38], [59], [62], [63], we have developed a gradient elution method using 0.1% formic acid in water (mobile phase A) and in methanol (mobile phase B) with the addition of 10 mM ammonium formate as the buffering agent. Other alternative mobile phases including acetonitrile were also evaluated but in the current study the separation was not enhanced. So we used acidified water and methanol to restrain the dissociation of acidic vitamins like nicotinic acid, pyridoxine, pantothenic acid and folic acid to promote improved ionization of the basic sites of all vitamins and moreover, to improve the peak shapes with higher resolution [64]. Different concentrations of formic acid and acetic acid (0.1–0.3%) were tested and 0.1% formic acid provided better peak shapes than acetic acid. Satisfactory resolution between peaks and excellent peak shapes were obtained for all the studied compounds under these RRLC analytical conditions that can be observed in Fig. 3 and Fig. 4.

The complete method of RRLC-MS/MS conditions for the analysis of water- and fat-soluble vitamins was optimized and we aimed at studying the vitamin profile of the phytococktail with this method. As described earlier, the phytococktail was prepared from three different plants of the trans-Himalaya and thus it could be considered as a complex nutraceutical with highly complex food matrices that increased our difficulties in estimating the complete vitamin profile of this phytococktail. It was observed that among the fat-soluble vitamins, α-tocopherol (E) was present in good amount. Among the water-soluble B-group vitamins, nicotinamide (B_3_), pantothenic acid (B_5_) and pyridoxine (B_6_) were found to be present in higher quantity as compared to thiamine (B_1_), riboflavin (B_2_) and nicotinic acid (B_3_). Pantothenic acid (B_5_) was the richest in all the studied water-soluble vitamins. The presence of highly abundant B-group vitamins in the phytococktail signifiy the mechanism of nutrient production in the rhizosphere by the root system in response to the highly stressful, fragile and nutrient deficient soil of the trans-Himalayan cold desert. The richness in vitamin E could possibly be due to the up regulation of the antioxidant defense mechanism of plants to combat the severe environmental stresses that was responsible for the production of several secondary metabolites and antioxidant compounds.

Amino acids are a class of biologically active compounds present in food and beverages which play an important role in human nutrition [65]. It also have an influence on the taste, aroma and color of natural foods, fruit based products and beverages [66], [67]. Hence, development of a reliable, rapid and precise method of amino acid analysis to evaluate the quality of foods and botanical products for nutritional and regulatory principles is of prime interest. Separation of amino acids by RP-HPLC with pre-column derivatization is a well established method for its simplicity, sensitivity and speed of separation [68]. In the present study, we have used the Pico-Tag method with PITC derivatizing reagent which was highly sensitive and capable of detecting nanogram (ng) amounts of amino acids in the food sample. Sea buckthorn was found to be a good source of proteins and free amino acids. A total of 18 amino acids were found in sea buckthorn fruit among which aspertic acid, threonine, proline, serine, lysine, valine and alanine were abundant [69]–[72]. Along with sea buckthorn, apricot fruits were also reported to be rich in amino acids. A recent study was carried out with 239 apricot cultivars from Czech Republic that revealed presence of 13 biogenic alpha-L-amino acids (arginine, asparagine, isoleucine, lysine, serine, threonine, valine, leucine, phenylalanine, tryptophan, tyrosine, proline and alanine) determined by ion exchange chromatography with UV-Vis spectrometry detection [73]. In another investigation, the amino acids of apricot fruits were separated and quantitatively determined by paper chromatography. The analysis revealed the presence of 19 amino acids, of which, glutamic acid, aspartic acid, histidine, arginine and tyrosine were predominant and the concentration of cystine, methionine and hydroxyproline was relatively small [74]. Separation and quantification of amino acids in apricot was also achieved by GC-MS [75]. Studies conducted with Italian commercial apricot juices by HPLC analysis revealed that asparagine was the major amino acid found in apricot juice (range 820–1570 mg/l), representing up to 78% of the total amino acid content and was also considered to produce an ‘intermediate extent of browning’ in the juice, whereas the most reactive amino acids were lysine, glycine, tryptophan and tyrosine [76]. There is dearth of information on the amino acid profile of *Rhodiola* sp. from all over the world. The amino acid profile of six *Rhodiola* sp. L. root and rootstalk from Xingjiang, China revealed the presence of 8 to 18 kinds of amino acids, of which 3 to 7 types were essential amino acids. *Rhodiola rosea* L. contained most classes of amino acid among the six *Rhodiola* sp. in Xingjiang, which was approved to be included as herbal drug in the traditional medicinal system of China [77]. Although the root of *R. imbricata* has been studied extensively for its pharmacological and therapeutic potentials, data on its free amino acid composition is not available till date. In the phytococktail, sea buckthorn was the prime ingredient and the other two plants were used to enhance its adaptogenic properties and organoleptic acceptance along with other health benefits. We had previously estimated the antioxidant potential of the phytococktail [39], [40] and thenceforth it was our primary interest to determine the diverse group of free amino acids present in the phytococktail that could be partly responsible for its bio-activities. In the present study, the phytococktail was found to be a rich source of diverse essential and non-essential amino acids which will be extremely beneficial for improving human health.

Our next aim was to estimate the fatty acid content of phytococktail. It was found to contain a number of diverse fatty acids. Survey of literature on the fatty acid composition of sea buckthorn growing in different parts of the world has revealed that the berries of this plant is rich in palmitic acid, palmitoleic acid, oleic acid and linoleic acid. The mesocarps of sea buckthorn principally contained palmitoleic acid (47.8%), palmitic acid (29.3%) and linoleic acid (10.6%). The phospholipids obtained from the berries of sea buckthorn mainly composed of palmitic acid (31.88%), oleic acid (26.21%) and palmitoleic acid (23.02%) [78]–[83]. The soft parts of the berries have a distinct fatty acid composition from that of the seeds. There is a large variation in the fatty acid composition of the sea buckthorn pulp, seed and oil depending on the subspecies, origins, cultivation activities, harvesting time of the berries and the extraction method. It contained high level of rare and valuable palmitoleic acid (16∶1 n-7, 16–54%), which is a component of skin fat and is known to support skin health and wound healing [81], [84]. Palmitic (17–47%) and oleic (2–35%) acids are the other two major fatty acids present in the soft parts [79]–[81], [84], [85]. These results are in agreement with our results obtained for the phytococktail where *H. rhamnoides* berries were used as the main ingredient. Apricot fruit was also used in the formulation of the phytococktail. Previous reports showed that the apricot fruits are a rich source of soluble sugars, protein, oil, fiber and fatty acids [86]–[90] as well as a diverse array of bio-active principles and minerals [91]–[95]. This fruit and other products based on it were found to possess a wide range of biological properties including antimicrobial, antimutagenic, tyrosine enzyme inhibition, cardioprotective, hepatoprotective, anti-inflammatory, antinociceptive and antioxidant activities [35], [39], [40]. However, the fatty acid composition of apricot fruit pulp was inadequately investigated. Most of the studies were performed with the kernel and seed oil and the major fatty acids were found to be palmitic acid (5.2%), oleic acid (61.4%) and linoleic acid (26.6%) [90]. There is lack of information on the fatty acid composition of *R. imbricata* root found in the trans-Himalayan region of India. Most of the studies were carried out on the essential oil composition of other *Rhodiola* sp. [96]. Hence, it limits our scope in comparing the results with previous reports on the fatty acid composition of *R. imbricata.*

High saturated fats and very low MUFA and PUFA have cholesterol and triacylglycerol lowering properties [97]. Conversely, high MUFA containing diets were also known for cholesterol lowering capacities [98], [99]. Elevated saturated fat (∼50%) in palm oil has reported to be suitable for the development of food products [100]. From our result, it was evident that palmitic acid was the only saturated fatty acid present in the phytococktail with significant amount. Studies conducted by previous investigators revealed that the total cholesterol (TC) and low density lipoprotein cholesterol (LDL-C) increasing properties of palmitic acid were lower in vegetable sources than animal sources [101]–[103]. On the other hand, palmitoleic acid was the dominant contributor of the total lipid content in the phytococktail. It is a less explored omega-7 fatty acid and rare to the plant kingdom. It has multifaceted actions in human health that includes cardiovascular and gastro-intestinal health, weight management, skin protection and anti-melanogenic properties [81], [104]–[107]. Research on palmitolec acid also revealed that it could avert beta-cell apoptosis [108], [109] and palmitoleic acid rich diets can improve circulating lipid profile [110]–[113]. Another major constituent of the total lipid content was oleic acid that also had important function in biological system [114]–[116]. Hence, the present findings support the medicinal and health-promoting potential of the phytococktail developed from the native trans-Himalayan plants.

## Conclusion

To the best of our knowledge, this is the first attempt towards the determination of nutritional profile of the phytococktail developed from the native trans-Himalayan plants. Extensive characterization of water- and fat-soluble vitamins in the phytococktail was carried out with RRLC-tandem mass spectrometry. The proposed process was simple, low-cost and rapid, with high sensitivity, reproducibility, robustness and time-efficiency which can be useful in the determination of vitamins in a variety of food supplements, pharmaceutical preparations, fortified beverages and complicated food matrices. The free amino acid profile of the phytococktail revealed the presence of 8 essential amino acids that are important for human nutrition. The phytocoktail was also found to contain substantial amount of omega-7 MUFA (palmitoleic acid), omega-9 MUFA (oleic acid), SFA (palmitic acid) and small quantity of carboxylic acid (linoleic acid), suggesting that the phytococktail could become an important natural source of fatty acids suitable for health promotion and disease prevention. The phytococktail could thus be considered as a prospective functional food that may offer potential health benefits. The information of nutritional profile in the phytococktail holds great significance from both dietary and nutritional aspects of human in the extreme high altitude environment. Finally, it can be concluded that the phytococktail could be a high value nutritional phytofood as it contains multiple vitamins, essential amino acids, fatty acids and dietary minerals to provide great health benefit.
